# Painful and Painless Diabetic Neuropathies: What Is the Difference?

**DOI:** 10.1007/s11892-019-1150-5

**Published:** 2019-05-07

**Authors:** Pallai Shillo, Gordon Sloan, Marni Greig, Leanne Hunt, Dinesh Selvarajah, Jackie Elliott, Rajiv Gandhi, Iain D. Wilkinson, Solomon Tesfaye

**Affiliations:** 1 Diabetes Research Unit, Royal Hallamshire Hospital, Sheffield Teaching Hospitals NHS Foundation Trust, Glossop Road, Sheffield, S10 2JF UK; 2 Department of Oncology and Human Metabolism, University of Sheffield, Sheffield, UK; 3 Academic Unit of Radiology, University of Sheffield, Sheffield, UK

**Keywords:** Diabetes, Peripheral neuropathy, Neuropathic pain, Small fiber neuropathy, Painful diabetic neuropathy, Diabetic neuropathy

## Abstract

**Purpose of Review:**

The prevalence of diabetes mellitus and its chronic complications are increasing to epidemic proportions. This will unfortunately result in massive increases in diabetic distal symmetrical polyneuropathy (DPN) and its troublesome sequelae, including disabling neuropathic pain (painful-DPN), which affects around 25% of patients with diabetes. Why these patients develop neuropathic pain, while others with a similar degree of neuropathy do not, is not clearly understood. This review will look at recent advances that may shed some light on the differences between painful and painless-DPN.

**Recent Findings:**

Gender, clinical pain phenotyping, serum biomarkers, brain imaging, genetics, and skin biopsy findings have been reported to differentiate painful- from painless-DPN.

**Summary:**

Painful-DPN seems to be associated with female gender and small fiber dysfunction. Moreover, recent brain imaging studies have found neuropathic pain signatures within the central nervous system; however, whether this is the cause or effect of the pain is yet to be determined. Further research is urgently required to develop our understanding of the pathogenesis of pain in DPN in order to develop new and effective mechanistic treatments for painful-DPN.

## Introduction

The worldwide prevalence of diabetes mellitus (DM) has reached epidemic proportions, and is set to increase to 629 million by 2045 [1]. Rising population growth, aging, urbanization, and an increased prevalence of obesity and physical inactivity are amongst the major contributing factors. Diabetic neuropathies are one of the most common chronic complications of DM [2], and distal symmetrical polyneuropathy (DPN) is the most prevalent form of diabetic neuropathy, which may affect up to 50% of patients [2, 3•, 4•]. The Toronto Expert Group has defined DPN as “a symmetrical, length dependent sensorimotor polyneuropathy attributable to metabolic and micro-vessel alterations as a result of chronic hyperglycaemia exposure and cardiovascular risk covariates” [5•]. A more recent definition of DPN in the American Diabetes Association Position Statement is “the presence of symptoms and/or signs of peripheral nerve dysfunction in people with diabetes after the exclusion of other causes” [3•]. The rising numbers of patients diagnosed with neuropathic disorders related to DM will have an immense impact on health and social care provision [6].

DPN is a major risk factor for diabetic foot ulceration, which remains a major cause of morbidity and is the leading cause of non-traumatic amputations [7]. Although a large number of patients with DPN may be entirely asymptomatic, approximately 15–25% of people with DM present with neuropathic pain (painful-DPN) [8–11, 12•, 13]. The neuropathic pain is of varying degree of intensity [14] DPN and painful-DPN has different clinical syndromes with the most common of which is a mixed large and small fiber neuropathy. Small nerve-fibers (SF) are small-caliber sensory fibers, which are primarily responsible for peripheral nociception [15]. Pure SF neuropathy may occur in DM and the clinical features include symptoms of painful peripheral neuropathy with signs of SF impairment (e.g., pinprick or thermal hypoalgesia or allodynia) in a peripheral neuropathy distribution in the absence of large fiber impairment (e.g., impaired light touch, vibration, proprioception or motor signs).

**Fig. 1:**
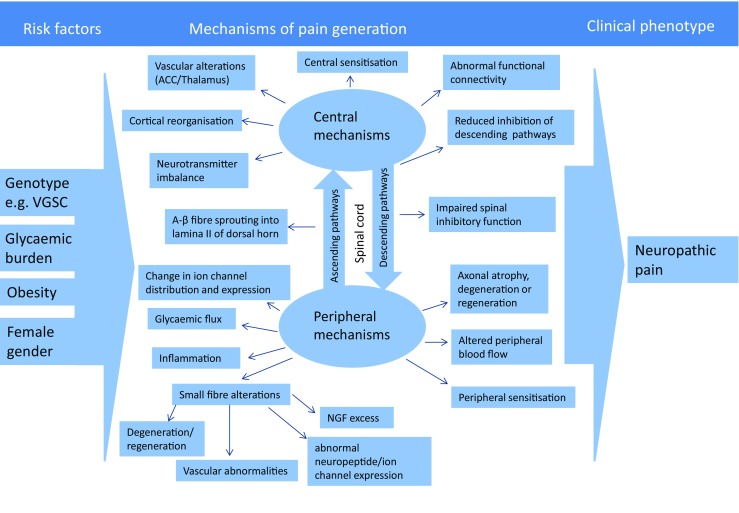
An overview of the current postulated pathogenesis of painful-DSPN. The risk factors for the generation of neuropathic pain in DSPN may include glycemic burden (duration of diabetes), obesity, female gender, and genetic variants of voltage-gated sodium channels (VGSC). Both the central and peripheral mechanisms have been postulated in the pathogenesis of painful-DSPN. ACC, anterior cingulate cortex. (Adapted from: Sloan G, et al. Diabetes Res Clin Pract. 2018; 144: 177–91, with permission from Elsevier) [17]

Painful-DPN often results in insomnia, mood disorders, and a poor quality of life [12•]. The currently available therapies for the pain associated with DPN remain inadequate, given relatively modest pain relief and often troublesome side effects [3•, 16, 17]. There is thus an urgent need to have a better understanding of the pathogenesis of pain in DPN and this has been the subject of a recent review (Fig. 1) [17]. Central to this understanding will be to develop new insights as to why some patients develop disabling neuropathic symptoms while others with a similar degree of neuropathy do not. This review will discuss the differences in risk factors, clinical features, serum biomarkers, vascular alterations, quantitative sensory testing (QST), skin biopsy parameters, genetics, and brain imaging studies between painful- and painless-DPN.

## Risk Factors

Several risk factors for DPN in general have been described and confirmed in cohorts of type 1 and type 2 diabetes. The EURODIAB Prospective Complications Study screened 3250 type 1 DM patients at baseline and followed 1172 patients without DPN looking for risk factors that predicted the development of DPN [4•]. The study found that in addition to glycemic control, traditional vascular risk factors such as hypertension, raised triglycerides, obesity, and cigarette smoking were independent risk factors for the development of new onset DPN. Similar vascular risk factors were also found in T2DM [18•, 19, 20]. However, the risk factors for neuropathic pain in DM are less well known. This is partly because of the wide variation in the diagnostic and population selection methods employed by the epidemiological studies for painful-DPN [21••, 22]. The reported risk factors include increasing age [9, 10], elevated HbA1c [23••, 24], duration of DM [9], and obesity [10, 25]. A high alcohol intake, type of diabetes, macro and microvascular disease, and ethnicity have also been implicated [21••]. Recent large studies have also suggested nephropathy and female gender as risk factors for painful-DPN [26, 27••, 28••]. Indeed, female gender was the only risk factor identified in a large cross-sectional study (*n* = 816) performed by Truini et al. which diagnosed painful-DPN using widely agreed criteria [28••]. Thirteen percent were diagnosed with painful-DPN and the only distinguishing risk factor from painless-DPN was female gender. Gender differences are well recognized in chronic pain conditions and neuropathic pain intensity has previously been reported to be more severe in females [29, 30].

Recent advances in gene sequencing technology have led to several studies examining genetic variants associated with DPN and painful-DPN [31–34, 35•, 36]. Two recent studies by Meng et al. conducted genome-wide association studies in Tayside, Scotland [32, 33]. Chr8p21.3, Chr1p35.1, and Chr8p21.3 polymorphisms were associated with neuropathic pain. However, the study did not use validated diagnostic criteria for painful-DPN. Recently also, there has been great interest in the role of voltage-gated sodium channels and their role in neuropathic pain. The Na_v_ 1.7 sodium channel is well recognized to be involved in pain signaling and “gain of function” mutations of its encoding gene, SCN9A, cause rare pain disorders. Additionally, studies have identified Na_v_ 1.7 mutations in idiopathic small fiber neuropathy [36] and painful-DPN [34]. Blesneac et al. looked at the relationship between Na_v_ 1.7 variants and painful-DPN and found that none of the participants with painless-DPN (*n* = 78) were found to have a genetic variant [35•]. However, a total of 12 rare Na_v_ 1.7 variants were identified in 10 out of 111 patients with painful-DPN. The subjects with these variants were found to have a shorter duration of diabetes yet more severe burning pain. Painful-DPN is a heterogeneous condition and subjects with rare sodium channel gene variants may represent a subgroup that may respond to a particular treatment.

In summary, while the risk factors for DPN are well recognized, those for painful-DPN are less certain. This might indicate the complexity of painful-DPN as many factors including genetics, cultural, psycho-social, and gender may be involved.

## Clinical Features

Neuropathic pain in diabetes has distinct presentations as burning, sharp, aching, electric, and evoked pains [37]. However, patients may also describe symptoms of numbness, tingling, and pins and needles, irrespective of the presence of pain. Neuropathic pain may also induce various degrees of physical disability, depression, anxiety, insomnia, and a poorer quality of life than patients with painless-DPN, particularly with moderate to severe neuropathic pain [23••, 27••, 38]. Despite these profound differences in a patient’s clinical presentation, there are few distinct differences in the neurological examination between painless- and painful-DPN. The majority of patients with painful-DPN demonstrate sensory loss on clinical examination but a small proportion of patients with painful-DPN have evidence of “gain of function” signs such as allodynia and hyperalgesia [23••]. There is controversy regarding whether the severity of neuropathic impairment is greater in painful-DPN. Several studies have reported a correlation between neuropathy severity and the presence and/or severity of neuropathic pain in DPN [8, 11, 15, 23••, 39–41] whereas other studies have not [18•, 28••]. Although the weight of evidence seems to suggest that an increasing severity of DPN may increase the risk of developing painful neuropathic symptoms, severe DPN and pain are not mutually exclusive, and there may have been a selection bias in recruiting painful-DPN patients from tertiary referral centers.

## Cardiovascular Autonomic Neuropathy

Both autonomic neuropathy and painful-DPN involve small fibers, and a potential relationship was therefore investigated. In a small study, we demonstrated greater changes in heart rate variability studies, as measures of cardiovascular autonomic neuropathy (CAN) in subjects with painful- compared with painless-DPN [42], while other small studies reported that painful DPN was more likely to be associated with the absence of a nocturnal fall in blood pressure (“non-dipping”) [43], or with reduced Valsalva ratio [40]. However, these are in contrast with other studies that have not found any differences in measures of CAN between painful and painless-DPN [39, 44, 45].

### Diagnostic Methods of Painful-DPN

Conventional neurophysiological testing methods, which measure large fiber function, such as nerve conduction studies (NCS), cannot detect pure small fiber neuropathy (SFN) [46]. However, QST and more recent advances in diagnostic techniques, e.g., skin biopsy with intraepidermal nerve fiber density (IENFD) quantitation, corneal confocal microscopy (CCM), and laser Doppler imaging flare (LDI Flare) have allowed the reliable diagnosis of SFN [5•, 46]. Because of their role in physiological nociception, studies have explored whether damage or alterations in SF may relate to neuropathic pain in DPN.

#### Skin Biopsy

Immunostaining of skin biopsy samples with protein gene product 9.5 and quantitation of IENFD is a reliable means of diagnosing SFN [46]. However, IENFD is unable to distinguish between individuals with or without neuropathic pain [23••, 27••, 28••, 47••, 48, 49]. Other studies have been performed to determine whether morphological and functional markers of the epidermal innervation revealed differentiating features. Intraepidermal nerve fiber (IENF) regeneration, by measuring the ratio of growth associated protein-43 (GAP-43) to nerve fibers, has been shown to be enhanced in painful-compared with painless-DPN [49, 50•, 51]. However, Scheytt et al. found no relationship between pain and GAP-43 reactivity in subjects with peripheral neuropathies of varying etiologies [52]. There are contradictory findings in studies investigating other IENF markers to differentiate painful- from painless-DPN including IENF length [50•, 53] and axonal swellings, which are measures of axonal degeneration [49, 54]. Levels within the skin of the neurotrophin nerve growth factor (NGF) were increased in patients with DPN and sensory symptoms, including pain, compared to painless-DPN [55]. NGF has recently been shown to sensitize nociceptors in human skin and it has been hypothesized that the remaining IENF in painful-DPN may be exposed to excessive levels of NGF (“over-trophing”) resulting in hypersensitivity and neuropathic pain [19, 55–58].

#### Corneal Confocal Microscopy

Confocal corneal microscopy (CCM) can rapidly, non-invasively, and accurately image corneal nerves and is a recently developed diagnostic test for DPN [59–61]. Studies of CCM have explored the role of corneal innervation and neuropathic pain in DM [53, 62•, 63]. Quattrini et al. reported reduced corneal nerve fiber length with unaltered other CCM measures [53], whereas Marshall et al. found unaltered corneal nerve fiber length but reduced corneal nerve fiber density [62•]. Recently, Kalteniece et al. [63] described significantly lower corneal inferior whorl length, and average and total nerve length in painful- compared to painless-DPN. Changes within this region have been suggested to be indicative of early neuropathic damage. However, there were confounding factors, which could account for these group differences. Therefore, the association of CCM abnormalities to neuropathic pain in DPN is thus far inconclusive.

#### Evoked Responses

Non-invasive tests have been developed to investigate the peripheral function of SF to diagnose SFN. Such tests can measure evoked potentials in response to stimuli that activate the nociceptive pathway, for example contact heat-evoked potentials (CHEPS) [64]. CHEPs correlates with other measures of SFN including IENFD and leg skin flare responses [65, 66]. One small study found a relationship between enhanced brain CHEP amplitudes in subjects with painful-DPN; this result was most marked in those with thermal hyperalgesia and mechanical allodynia [67].

#### Quantitative Sensory Testing

QST is a psychophysical measure of the perception of different external stimuli of controlled intensity to assess a range of sensory modalities [68, 69]. Some studies with a relatively small sample size suggested that conventional QST measures of SF function may be statistically different between painful- and painless-DPN [41, 44, 70, 71]. More recent studies have employed the German Research Network on Neuropathic Pain (DFNS) QST protocol to quantify sensory loss, for small and large fiber function, and sensory gain abnormalities [72, 73••]. Three recent large cross-sectional cohort studies have applied this protocol to patients with painful- and painless-polyneuropathies with different etiologies [48] and painful- and painless-DPN [23••, 27••]. In two studies of painful-DPN, DFNS QST revealed more severe loss of function in those with neuropathic pain, particularly patients with moderate/severe pain [23••, 27••]. Thermal hyposensitivity was more severe in painful-DPN whereas mechanical stimuli showed fewer differences compared with painless-DPN. Gain of function abnormalities and preserved SF function with hyperalgesia were both rare. However, Üçeyler et al. studied patients with painful- and painless-polyneuropathies of different etiologies and found that patients with neuropathic pain demonstrated elevated mechanical pain and detection threshold, and lower mechanical pain sensitivity with no difference in SF deficits [48]. This perhaps indicates there may be a unique somatosensory phenotype associated with painful-DPN characterized by more severe SF dysfunction with thermal hyposensitivity [23••, 27••]. However, SF changes are common and can occur in early DPN without pain [74–76]; therefore, these findings alone are unable to completely explain why some patients develop neuropathic pain and others do not. Perhaps, other investigations into small fiber function and structure, such as skin biopsy studies, may shed further light onto this paradox.

## Pathogenesis of Painful-DPN

### Microvascular Blood Flow

Consistent with vascular risk factors increasing the risk of DPN [4•], both structural and functional microvascular abnormalities of the vasa-nervorum have been shown to be involved in the pathogenesis of DPN [77–79]. Patients with treatment induced neuropathy of diabetes who had extremely severe neuropathic pain have proliferating blood vessels on the epineurial surface bearing striking similarities to those found in proliferative diabetic retinopathy [80]. It is well recognized that very rapid improvement in glucose control can cause proliferative retinopathy mediated by retinal ischemia and a similar process appears to take place in the peripheral nerve. Furthermore, several studies have shown that regulation of peripheral blood flow is altered in patients with painful- compared with painless-DPN [81–84]. Our group demonstrated elevated sural nerve epineurial oxygen saturation and faster blood flow in patients with painful- compared to painless-DPN, perhaps secondary to arteriovenous shunting [82]. Other studies have examined the role of skin microvascular vasodilator and vasoconstrictor responses in subjects with DPN, with contradictory findings [71, 85–87].

Studies measuring serum markers of angiogenesis (vascular endothelial growth factor, VEGF) and endothelial dysfunction (soluble intercellular adhesion molecule – 1, sICAM-1) have found them to be elevated in painful-DPN [86, 88•] and symptomatic DPN respectively [89]. Furthermore, punch skin biopsy studies have also indicated that skin microcirculation may be involved in the pathogenesis of painful-DPN. One study demonstrated evidence of hypoxia, by immunostaining with hypoxia inducible factor 1α (HIF-1 α), to be related to pain intensity in subjects with DPN [90]. Recently, our group has also found dermal von Willebrand factor (vWF) immunoreactivity, as a blood vessel marker, to be significantly elevated in subjects with painful-DPN, in comparison to subjects with painless-DPN, patients with DM without DPN and healthy volunteers [56]. Moreover, small studies have demonstrated that pain improves with topical application of vasodilator treatments [91, 92], perhaps indicating that local blood flow dysregulation could be a viable target for the management of pain in DPN.

### Hyperglycemia and Downstream Effects

Hyperglycemia mediated metabolic pathways have long been associated in the pathogenesis of DPN, but their role in those with neuropathic pain is less clearly defined. Studies using DM rodent models have found neuropathic pain behaviors to be related to numerous metabolic pathways including the polyol pathway, protein kinase C activity, and increased advanced glycation end-products (AGEs) [93]. However, there is limited evidence to support glycemic control or lifestyle modifications in improving painful neuropathic symptoms [3•]. Moreover, the evidence to support pathogenic treatments for neuropathic pain in DPN has generally been disappointing and only a few pharmacotherapeutic agents are available in select countries [50•].

Methylglyoxal is a highly reactive dicarbonyl compound and is a precursor to the formation of (AGEs). The formation of AGEs has downstream deleterious effects on peripheral nerves and Schwann cells including inflammation and oxidative stress [94]. Methylglyoxal has been suggested to be an important factor in the development of DM and incident DPN [20•, 95]. In rodent models of painful-DPN, methylglyoxal has been shown to induce hyperalgesia via activation of the voltage-gated sodium channel Nav 1.8 and transient receptor potential channel ankyrin-1 [96, 97]. Similarly, in a small number of patients with DM (*n* = 30), serum methylglyoxal levels were found to be elevated in painful-DPN [96]. In contrast to these findings, a larger study (*n* = 882) reported methylglyoxal levels to be unrelated to painful-DPN [98]. Although the role of hyperglycemia mediated pathways in generating neuropathic pain is uncertain, pathogenically oriented treatments, particularly anti-oxidants, have been demonstrated to improve pain in some pre-clinical and clinical trials [99, 100].

### Vitamin D

Although vitamin D is most commonly recognized for its role in calcium metabolism and bone health, vitamin D is involved in many disparate physiological processes [101]. Deficiency of vitamin D has been shown to be predictive of numerous chronic diseases including DM, DPN, and chronic pain [101–104]. Pre-clinical studies indicate that vitamin D appears to play a critical role in nerve function in health and may play a role in neuropathic pain syndromes [105–109]. Our group recently found vitamin D levels to be significantly lower in patients with painful- compared to painless-DPN, with a significant correlation between serum 25-hydroxyvitamin D level and pain scores on the Doleur Neuropathique 4 neuropathic pain screening tool [110•]. The study was cross-sectional, and therefore cannot establish a causal relationship, but it does suggest a possible mechanistic link between vitamin D and painful-DPN. Indeed, three non-randomized clinical trials have demonstrated an improvement in painful neuropathic symptoms with vitamin D therapy but, further, larger, adequately powered RCTs are necessary to investigate this further [111–113].

### Inflammation

Inflammation has been postulated to play a major part in DM and DPN [114]. Low grade inflammation has been suggested as a link between obesity and T2DM, via inflammation induced insulin resistance [114]. Inflammatory chemokine and cytokine production has been reported to be induced by several metabolic pathways implicated in the pathogenesis of DPN [115, 116•]. Multiple studies have demonstrated higher systemic acute-phase proteins, cytokines, and chemokines in DPN [88•, 117•], recently reviewed by Bönhof et al. [118•]. Furthermore, rodent models of neuropathic pain associated with the metabolic syndrome and T2DM demonstrate elevated pro-inflammatory mediator expression in the serum [119] and the dorsal root ganglia [119, 120]. The outcomes of studies examining the association of inflammatory biomarkers and painful-DPN have been variable. Numerous inflammatory markers have been associated with painful-DPN: C-reactive protein (CRP) [86], tumor necrosis factor-α (TNF-α) [121], inducible nitric oxide synthase [121], and interleukin 6 [117•]. Additionally, inflammatory mediators have been shown to differentiate between painful- from painless-neuropathies of various etiologies, including elevated serum IL-2, TNF-α, and reduced anti-inflammatory IL-10 [122]; IL-6 and IL-10 sural nerve biopsy expression [123]; and TNF-α in human Schwann cells [124].

### The Central Nervous System

Technological advances in imaging modalities have enabled detailed in vivo investigation of the nervous system in DM. Key differences have been identified within the CNS in painful-DPN, using a variety of different techniques, especially advanced MR imaging modalities.

### Spinal Cord Changes in Painful-DPN

We have identified a reduction in the cross-sectional area of the spinal cord in subjects with DPN in comparison to patient with DM without DPN, healthy controls, and disease control subjects with hereditary sensory motor neuropathy type 1A [125]. However, structural differences in the spinal cord area have not been found between subjects with painless- and painful-DPN [125]. Recent studies have indicated that spinal disinhibition, measured using the rate dependent depression (RDD) of the Hoffman reflex (H-reflex), may be a potential biomarker of spinally mediated pain to differentiate painful- from painless-DPN [126]. The RDD has been demonstrated to assess γ-aminobutyric acid (GABA) type A receptor-mediated spinal inhibitory function in neuropathic pain models of DM rats [127]. Impaired RDD was found in DM rat models of T1DM and T2DM with neuropathic pain phenotypes [62•]. Also, the RDD in groups of healthy controls and T1DM subjects with painful- and painless-DPN was evaluated and it was significantly impaired in those with painful-DPN. Patients with greater RDD attenuation had higher pain scores but no difference in measures of large or small fiber dysfunction, perhaps suggesting spinal inhibitory dysfunction may occur independent of PNS alterations in painful-DPN.

### Advanced MRI Studies of the Brain

Functional MRI (fMRI) measures the activity of brain regions by detecting changes in the oxygenation of hemoglobin, the blood oxygen level dependent signal (BOLD). The neurological signature of physical pain has been identified by fMRI and includes activation of the venterolateral thalamus, dorsal posterior insula, and somatosensory cortex, as well as brain regions related to emotional pain processing, including the anterior insula and anterior cingulate cortex (ACC) [128–130].

### The Thalamus

The thalamus receives somatosensory signals from the spinal cord where they are processed, modulated, and transmitted to higher brain centers. A variety of brain alterations have been demonstrated in DPN and painful-DPN using advanced imaging techniques that examine brain neurochemistry, microvascular blood flow, and functional changes. MR spectroscopy (MRS) enables the measurement of selected metabolites within the brain [131]. Our group has used MRS to show neuronal dysfunction within the thalamus, by reduced N-acetyl aspartate (NAA) to choline ratio as a neuronal marker, in subjects with painless-DPN [132]. Furthermore, DM animal models of painful-DPN have shown that the thalamus may be responsible for central amplification of somatosensory signals [133, 134]. Similarly, thalamic dysfunction appears to play a key role in human painful-DPN. We have recently shown preserved thalamic NAA and the GABA levels within the thalamus in patients with painful-DPN, whereas these levels were reduced in patients with painless-DPN [135, 136]. These findings suggest that neurochemical measures of the thalamic neuronal function and neurotransmitters may be essential for pain signal transmission and/or amplification in painful-DPN.

Recently, we performed a study administering exogenous perfusion contrast to subjects with painful- and painless-DSPN to compare the thalamic microvascular perfusion at rest [137•]. Subjects with DPN both demonstrated delayed bolus arrival time to the thalamus, but subjects with neuropathic pain had a significantly taller peak concentration, a higher mean cerebral blood volume, and the longest blood transit time compared to painless-DPN. Therefore, microvascular vasodilation within the thalamus may induce hyperperfusion which could be related to elevated thalamic neuronal activity. Finally, fMRI study of the brain has indicated there may be disruption in thalamocortical connectivity in painful-DPN. Cauda et al. measured resting state fMRI to determine temporal correlations of brain activity in a small number of subjects with painful-DPN and healthy control patients [138]. Compared with the control group, there was reduced synchrony between the somatosensory cortex and thalamic nuclei in painful-DPN patients.

### Descending Inhibition

It is well recognized that the midbrain and medullary brain regions can exert bidirectional control over nociception [139]. The periaqueductal gray (PAG) and rostroventromedial medulla (RVM) are key sites for the control of descending pain modulation, disruption of which in rodent models of painful-DPN has been shown to lead to enhancement of descending pain facilitation [140, 141]. In human studies, our group performed MR-dynamic susceptibility contrast imaging at rest and under experimental pain, by applying heat pain to the lateral thigh where participants did not experience neuropathy [142]. During experimental pain, the time to peak concentration of contrast reduced in healthy volunteers but significantly increased in subjects with painful-DPN in the bilateral sensory cortices and thalami, perhaps indicating an underlying impairment in descending inhibition. Segerdahl et al. interrogated the ventrolateral PAG (vlPAG) using resting state fMRI and arterial spin labelling to determine cerebral blood flow at rest and during heat stimulation to the foot [143•]. The painful-DPN group demonstrated altered vlPAG functional connectivity, which correlated to their pain intensity and the cerebral blood flow changes induced by experimental thermal stimulation. These studies indicate that abnormalities within the descending pain modulatory system may result not only in reduced inhibition of pain but increased amplification of pain signals in painful-DPN.

### Higher Brain Centers

The higher brain centers are involved in the localization of pain (e.g., somatosensory cortex) as well as the behavioral, cognitive, and emotional response to painful stimuli (e.g., ACC, amygdala, insular cortex). Using a technique known as voxel-based morphometry, we calculated the brain volumes in subjects with DPN and identified total brain volume reduction which was localized to the somatosensory regions [144, 145]. Furthermore, our group has performed the largest cohort study of brain volume changes in DPN and painful-DPN to date [146]. In painful-DPN, cortical atrophy is localized within the somatomotor cortex and insula. We have also demonstrated abnormal cortical interactions within the somatomotor network at rest which correlated with measures of pain and behavior in subjects with painful-DPN [147]. A recent study performed single-photon emission computed tomography to assess cerebral blood flow (CBF) in 24 subjects with painful- and 20 painless-DPN [148]. The painful-DPN group demonstrated increased CBF within the right ACC and left nucleus accuumbens. However, the painless-DPN group demonstrated more severe neurophysiological neuropathic impairment which may be a potential confounding factor. Furthermore, application of thermal heat stimuli resulted in altered BOLD fMRI responses in painful- compared with painless-DPN, seen in two studies [149, 150]. A pilot study within our group found greater BOLD response within the primary somatosensory cortex, lateral frontal, and cerebellar regions [149]. Whereas Tseng et al. demonstrated augmented responses in multiple limbic and striatal structures (i.e., ACC, superior frontal gyrus, medial thalamus, anterior insular cortex, lentiform nucleus, and premotor area) with the BOLD signal in the ACC and lentiform nucleus correlating with pain rating to thermal stimulation [150]. It is currently unknown whether the CNS changes described in these studies are a response to peripheral nervous system afferent inputs or a primary mechanism responsible for the maintenance of neuropathic pain.

## Conclusions

Painful-DPN is a major cause of morbidity in patients with DM. Unfortunately, our understanding of why patients with DPN develop neuropathic pain remains inadequate (Fig. 1.). We have summarized the current evidence of the differences between painful- and painless-DPN (see Table 1.). However, there are limitations in many of the studies including small sample sizes, inappropriate definition of neuropathic pain and DPN, and measurement of multiple variables, leading to a risk of false positives. More recently, large, well-characterized cross-sectional cohort studies have given valid insights into the risk factors and somatosensory profiles of painful-DPN [23••, 27••, 28••]. Unfortunately, longitudinal studies which prospectively identify definitive differences in painful-DPN have not yet been performed, and would be logistically challenging and costly to perform. These limitations notwithstanding, painful-DPN seems to be associated with female gender, increased small fiber injury and/or function, and peripheral/central vascular alterations. The role of autonomic dysfunction, vitamin D, inflammatory mediators, genetic factors, and methylglyoxal needs further clarification. Studies of the CNS demonstrate clear differences in painful- compared with painless-DPN. The spinal, somatomotor, limbic, thalamic, and ascending and descending modulatory systems demonstrate alterations using numerous testing techniques. However, what remains unclear is the causal relationship between painful-DPN and CNS changes. Further studies are necessary to determine whether these findings are the primary cause of neuropathic pain or adaptive to neuropathic afferent impulses. Irrespective of this, advanced MR imaging modalities have the potential for acting as biomarkers for monitoring therapeutic responses to treatments [151••].

**Table 1:** Recently reported differences between painful- and painless-diabetic peripheral neuropathy

Contributing factor	Difference associated with painful-DPN	References
Risk factors	Female gender	[21••, 26, 27••, 28••]
	Nephropathy	[26, 27••]
	Na_v_ 1.7 mutations	[35•]
Small nerve fiber alterations	Hyposensitivity phenotype	[23••, 27••]
	Epidermal nerve fiber regeneration	[49, 50•, 51]
Microvascular alterations	Elevated immunostaining for blood vessels	[56]
Vitamin D	Reduced 25-hydroxyvitamin D levels	[110•]
Inflammatory biomarkers	C-reactive protein, tumor necrosis factor-α, inducible nitric oxide synthase and interleukin 6.	[86, 117•, 121]
Central nervous system		
Spinal cord	Impaired spinal inhibitory function	[62•]
Thalamus	Preserved thalamic NAA and GABA neurochemistry	[135, 136]
	Thalamic hyperperfusion	[137•]
	Altered somatosensory cortex and thalamic functional connectivity	[138]
Descending modulatory pain centers	Descending pain facilitation	[142, 143•]
Higher brain centers	Somatomotor cortex and insula cortical atrophy	[146]
	Abnormal cerebral blood flow at rest and in response to heat pain	[142, 148]
	Altered functional connectivity in higher brain centers at rest and experimental pain conditions	[147, 149, 150]

*DPN* diabetic distal symmetrical polyneuropathy, *NAA* N-acetyl aspartate, *GABA* γ-aminobutyric acid, *BOLD*
